# What can mirror-touch synaesthesia tell us about the sense of agency?

**DOI:** 10.3389/fnhum.2014.00256

**Published:** 2014-04-24

**Authors:** Maria Cristina Cioffi, James W. Moore, Michael J. Banissy

**Affiliations:** Department of Psychology, Goldsmiths, University of LondonLondon, UK

**Keywords:** agency, mirror-touch synaesthesia, self-other distinction, ownership, self-other representation

Sense of agency (SoAg) refers to the feeling of control over one's actions and forms an integral part of our cognitive and social lives (Moore and Fletcher, 2012). For example, it is thought that the recognition of oneself as the agent of an action plays a fundamental role in self-awareness (Jeannerod, 2003). It is also thought that the experience of agency is important for guiding our attributions of responsibility (Haggard and Tsakiris, 2009).

The importance of SoAg is also demonstrated by the striking changes in this experience associated with various psychiatric (e.g., schizophrenia) and neurological (e.g., cortico-basal degeneration) disorders. While in recent years a number of studies have examined SoAg in these clinical groups, one group of individuals that have not yet been examined are those with mirror-touch synaesthesia (MTS). This opinion article seeks to explain why changes in SoAg may occur in MTS and also why mirror-touch synaesthetes could offer unique insights into the neurocognitive basis of SoAg.

For most of us, observing another person being touched activates neural regions in the somatosensory cortex that are also involved in experiencing touch (e.g., Keysers et al., 2004, 2010; Ebisch et al., 2008; Schaefer et al., 2012), however this activation does not lead to overt sensations of the observed event: we typically do not feel any tactile sensation when observing the tactile experience of others. On the contrary, people with MTS, approximately 1.6% of the population (Banissy et al., 2009), do experience overt tactile sensations to the observed event: they feel tactile sensations on their body when simply seeing touch to another's body (Blakemore et al., 2005; Holle et al., 2011; Banissy, 2013). These experiences are reported to be automatic (Banissy and Ward, 2007), enduring (Holle et al., 2011), and may be associated with broader differences in social perception (Banissy and Ward, 2007; Banissy et al., 2011; Goller et al., 2013).

Recent studies (e.g., Aimola-Davies and White, 2013; Holle et al., 2013; Maister et al., 2013) suggest that individuals with MTS have atypical self-other representations. For example, Maister et al. (2013) ran a study using the “enfacement illusion” paradigm. In the typical “enfacement illusion,” participants are asked to say to what extent images of faces that were morphed between themselves or another person look like themselves or the other; they then watch a video in which the other person is touched in synchrony and congruent with a felt touch on the participant's face. After experiencing a synchrony between the observed and felt touch, the images that participants had initially perceived as containing equal quantities of self and other became more likely to be recognized as the self (Tsakiris, 2008; Tajadura-Jiménez et al., 2012). Maister et al. (2013) adapted this paradigm in MTS, by removing the physical touch component. That is to say that individuals observed touch to other people, but veridical synchronous touch was not physically applied to the face. They showed that MT synaesthetes experienced the same effect of “enfacement illusion” in the absence of a touch applied to their face, concluding that simply viewing the touch on others evokes changes in self-other representations in MTS. In this regard, MTS may therefore be characterized as bringing more malleable body representations, reflecting a blurring in the self-other distinction processes (Banissy and Ward, 2013; Maister et al., 2013).

This self-other blurring may be significant for SoAg. Experimental work in neurotypical individuals has shown how the deliberate blurring of the boundaries between self and other can have dramatic effects on SoAg. A good example of this is the so-called “Vicarious Agency” illusion, first demonstrated by Wegner et al. (2004). In this paradigm, participants sit in front of a mirror with their arms placed out of view, under a sheet that covers everything below their shoulders. A cardboard shield is placed behind their back to block their view of the experimenter standing behind them. The experimenter places their arm forward so that it appears where the participant's own arm would have been. This set-up is therefore aimed at engendering self-other confusion. Participants are then asked to look at the mirror in front of them, while the experimenter performs the gestures. Participants also wear headphones on which are played action previews (e.g., “wave your hand,” “make the ok gesture”). These previews are either congruent or incongruent with the actions subsequently made by the experimenter. Wegner et al. found that participants experienced a SoAg and ownership over the arm that appeared in the mirror and that their experience of controlling the movements was increased when the previews were congruent with the action the experimenter made. In this way, we can see how an experimentally-induced blurring of the boundaries between self and other has a striking effect on SoAg. A strong prediction from this finding is that individuals with MTS will be more vulnerable to these agency illusions. This is something we are currently testing.

Another line of enquiry worth pursuing is whether or not these putative agency effects in MTS are mediated by the changes in the sense of body ownership associated with the condition (e.g., Aimola-Davies and White, 2013; Maister et al., 2013). The sense of body ownership refers to the feeling that the body one inhabits is one's own. Importantly, the sense of body ownership and SoAg are not independent. For example, it is often assumed that SoAg is predicated on recognizing that the moving body part is one's own. The existing work on MTS would suggest that changes in sense of ownership represent a primary disturbance in the condition. One prediction, therefore, is that the putative changes in SoAg are a *consequence* of these fundamental disturbances in sense of ownership. Intriguingly, the relationship between agency and ownership can also work in the opposite direction. Previous research in neurotypical adults has shown that SoAg can play a role in structuring bodily awareness (e.g., Tsakiris et al., 2010). In the context of MTS, one prediction from this would be that if there were agency-processing deficits these would exacerbate more basic disturbances in bodily awareness. We are clearly suggesting here that MTS is primarily a “disorder” of ownership, which can have consequences for SoAg and which in turn can further worsen ownership disturbances. However, at present this is speculative and is something that should be systematically examined in future research.

A further benefit of examining of SoAg in MTS is that it may help constrain our understanding of how inter-individual differences in self-other representations involved in SoAg and sense of body ownership interact to structure bodily awareness. Indeed, it has been shown that patients with impairments in self-other discrimination perform poorly on agency tasks: in particular, Daprati et al. (1997) showed that people with schizophrenia had difficulties when required to correctly identify the origin of an action. Even in the absence of clinical implications, it is likely that individuals with MTS can experience a distortion in their SoAg and could be a non-clinical framework for studying the determinants of agency and its disruptions. It is in this context that MTS may also help inform models of SoAg, increasing our understanding of the interaction between ownership and agency. It is our contention that MTS offers a rare opportunity to investigate this interaction more directly.

In the context of existing models of SoAg there are also some specific predictions about agency processing in MTS that could be tested. For example, the so-called comparator model of SoAg (e.g., Blakemore et al., 2002) states that predicted sensory feedback is subtracted out of the actual sensory percept during movement. According to the model this sensory attenuation is a key mechanism that allows us to distinguish between self- and externally-generated effects. Previous work in neurotypical individuals has shown that sensory suppression is only found for self-generated movements and not when observing someone else move (Weiss and Schütz-Bosbach, 2012). However, given the disturbances in self-other discrimination in MTS one might predict that individuals with MTS would also show sensory suppression effects when observing someone else move.

The final benefit of research on MTS that we wish to highlight concerns the brain basis of SoAg. Although a great deal of work has been done on the neural correlates of SoAg, we still know relatively little about the neural *networks* and *mechanisms* underpinning it (see David, 2012, for a review). We would suggest that research on MTS could help in this regard by furnishing our understanding of the brain basis of SoAg. Two regions commonly implicated in SoAg are the anterior insula and temporo-parietal junction (TPJ). The anterior insula is heavily linked with self-other discrimination (Ruby and Decety, 2001) and is also activated in agency attribution tasks (e.g., Farrer and Frith, 2002). Concerning the TPJ, many studies on SoAg that rely on the comparison between self-generated and the externally produced sensory signals have found activation in the right TPJ (Ruby and Decety, 2001; Farrer et al., 2003; see Decety and Lamm, 2007 for a meta-analysis of fMRI studies on TPJ). Interestingly, the anterior insula and TPJ also appear to play a key role in MTS. A common suggestion is that MTS reflects a hyper-activation of the mirror-touch network; that is, brain regions involved in experiencing and passively observing touch to others, including the primary and secondary somatosensory cortices (SI, SII) (Blakemore et al., 2005; Holle et al., 2013). Banissy and Ward (2013), suggest that this hyper-activation of the mirror-touch system in individuals with MTS may be gated by atypical functioning in neural regions involved in self-other representations, and highlight potential roles for both the anterior insula and the TPJ in this process. One potential avenue for future research would be to examine whether this putative gating mechanism is functionally relevant for SoAg, perhaps having a role in modulating more basic sensorimotor processes known to be important for this experience.

In summary, MTS refers to a rare experience in which observing touch or pain to another person evokes a tactile experience on the observer's body. There is growing evidence to suggest that this is linked to a blurring of self-other representation. In this article we have discussed how this disturbance may produce changes in SoAg in MTS. We have also discussed the ways in which research on MTS can improve our understanding of the neurocognitive basis of SoAg. In light of these discussions we believe that future research on SoAg in MTS is likely to provide valuable insights, both for those with a primary interest in MTS and for those with a primary interest in SoAg.

## Conflict of interest statement

The authors declare that the research was conducted in the absence of any commercial or financial relationships that could be construed as a potential conflict of interest.
